# THE IMPACT OF THE OPIOID EPIDEMIC ON OLDER ADULTS: FINDINGS FROM A NATIONAL SURVEY OF COMMUNITY-BASED ORGANIZATIONS

**DOI:** 10.1093/geroni/igz038.2706

**Published:** 2019-11-08

**Authors:** Kathleen A Cameron, Lauren E Popham, Angelica Herrera-Venson

**Affiliations:** National Council on Aging, Arlington, Virginia, United States

## Abstract

The National Council on Aging (NCOA) conducted a national survey of community-based organizations (CBOs) in early 2019 to better understand how older adults, people with disabilities, and their caregivers are affected by the opioid epidemic and identify new resources and tools needed by CBOs to better serve their community needs. Specifically, the survey asked about the extent to which CBOs’ service delivery and level of effort has changed as a result of the opioid epidemic; unique issues reported by this population, directly or indirectly resulting from opioid misuse by them or loved ones; how organizations screen and refer older adults and individual with disabilities for support associated with opioid misuse; how organizations may be connecting with local or state initiatives addressing the opioid epidemic, or forming strategic partnerships to respond to emerging client needs; and pinpoint gaps in resources that may help organizations to more effectively respond to these issues. Over 200 organizations, representing urban, suburban and rural communities, responded to the survey and included senior centers, area agencies on aging, Senior Health Insurance Assistance Programs, as well as health care organizations. Seventy percent of organizations report spending more effort to address the needs of older adults/caregivers who are adversely affected by opioid misuse/abuse since 2 years ago. Common health and financial concerns, current strategies related to screening, partnership development, and educational programming as reported by CBOs will be presented. This session will include a discussion of opportunities to assist CBOs address the opioid-related needs of their older adult clients.

